# Recent Advances in the Photorefraction of Doped Lithium Niobate Crystals

**DOI:** 10.3390/ma5101954

**Published:** 2012-10-22

**Authors:** Yongfa Kong, Shiguo Liu, Jingjun Xu

**Affiliations:** 1 The MOE Key Laboratory of Weak-Light Nonlinear Photonics, TEDA Applied Physical School, Nankai University, Tianjin 300457, China; E-Mail: jjxu@nankai.edu.cn; 2 School of Physics, Nankai University, Tianjin 300071, China; E-Mail: nkliusg@nankai.edu.cn

**Keywords:** photorefraction, lithium niobate, dopants, optical damage

## Abstract

The recent advances in the photorefraction of doped lithium niobate crystals are reviewed. Materials have always been the main obstacle for commercial applications of photorefractive holographic storage. Though iron-doped LiNbO_3_ is the mainstay of holographic data storage efforts, several shortcomings, especially the low response speed, impede it from becoming a commercial recording medium. This paper reviews the photorefractive characteristics of different dopants, especially tetravalent ions, doped and co-doped LiNbO_3_ crystals, including Hf, Zr and Sn monodoped LiNbO_3_, Hf and Fe, Zr and Fe doubly doped LiNbO_3_, Zr, Fe and Mn, Zr, Cu and Ce triply doped LiNbO_3_, Ru doped LiNbO_3_, and V and Mo monodoped LiNbO_3_. Among them, Zr, Fe and Mn triply doped LiNbO_3_ shows excellent nonvolatile holographic storage properties, and V and Mo monodoped LiNbO_3_ has fast response and multi-wavelength storage characteristics.

## 1. Introduction

Holographic data storage promises to become the next-generation optical storage technology for many years [1,2,3]. However, this technology has still not matured to be fit for commercial application. The main obstacle is the lack of an ideal medium [4]. Laser-induced optical damage [5], later named also as photorefraction [6], was discovered in LiNbO_3_ and LiTaO_3_ crystals. This effect can be utilized as holographic storage, laser physics, information processing and calculation. It was reported that photorefraction can be enhanced by transition-metal ions, such as Fe, Cu, Mn, Ni, *etc.* [7,8], and among them, iron doped LiNbO_3_ (LN:Fe) has the best photorefractive properties, such as high diffractive efficiencies, high data storage density and long dark storage time. Though LN:Fe went on to be the mainstay of holographic data storage efforts, several problems, such as low response speed, strong light-induced scattering and volatility impede it from becoming a commercial recording medium.

As to the volatility, the read-out process usually erases the stored information and amplifies the scattered light. Several techniques for “fixing” holograms have been developed [9,10,11,12]. However, they have practical disadvantages, and only laboratory demonstrators have been built. It 1998, Buse *et al.* [13] used iron and manganese doubly doped LiNbO_3_ (LN:Fe,Mn) to form two different deep electron traps; illumination of the crystals with incoherent ultraviolet light during the recording process with a red-light interference pattern permits the storage of data that can be subsequently read without erasure in the absence of ultraviolet light. This resolution to the problem of volatility leads to the realization of a more practical system, but the response time is in the order of minutes, which is apparently too long for practical applications. Later, Cu and Ce codoped LiNbO_3_ (LN:Cu,Ce) was reported as another crystal that can be used for nonvolatile holographic storage [14]. Cu and Ce ions occur in the valence states Cu^+/2+^ [15] and Ce^3+/4+^ [16], respectively, which are regarded as shallow and deep centers [17]. However, the response time of LN:Cu,Ce is even longer than that of LN:Fe,Mn. To realize a real-time non-volatile read-write memory, shortening of the response time and improving the sensitivity seem to be the critical challenge.

One way to solve this problem is to increase the ratio of [Li]/[Nb], in other words, growing near-stoichiometric (NS) crystals [18]. However, this is difficult to achieve. Until now, even without dopants, strictly stoichiometric pure LiNbO_3_ ([Li]/[Nb] = 50/50) single crystals of practical size and quality have not been grown. As to doubly doped LiNbO_3_, we do not even know where the stoichiometric melt composition is. On the other hand, laser-induced optical damage of LiNbO_3_ can be greatly degraded by optical damage resistant additives, such as Mg, Zn, In and Sc [19,20,21,22], as soon as the doping concentration exceeds a certain threshold. LN:Fe crystals codoped with optical damage resistant elements have high resistance against light-induced scattering and fast response speed for concentrations above threshold, as has been demonstrated by Zhang *et al*. for LN:Mg,Fe [23]. The main mechanism of this phenomenon is that above the doping threshold, a part of the Fe^2+^ and all of the Fe^3+^ ions are repelled from Li-sites to Nb-sites, which causes an abrupt decrease of the capture cross-section of electrons by Fe^3+^ and, as a consequence, a sharp increase of photoconductivity and, therefore, a fast response speed. Nevertheless the decrease of Fe^2+/3+^ ions in Li-sites will cause an apparent decrease of the diffraction efficiency [24].

There are plenty of papers about the photorefraction of LiNbO_3_ crystals; for a detailed understanding of the background, one can see the books published by Springer [25,26]. In the past decade, there have also been many important reports on this topic, such as that optical damage can be strongly suppressed by optical cleaning [27]. It is not possible to cover in this brief article the immense variety of photorefraction in LiNbO_3_ and their experimental and theoretical studies. Here we just briefly reviewed some recent advances in the photorefraction of doped LiNbO_3_, mainly about the newly reported dopants in the last ten years. Our particular choice of topics does not reflect on the importance of those issues that are not discussed.

## 2. The Optical Damage Resistance of Tetravalent Ions Doped LiNbO_3_

When Kokanyan *et al.* [28] grew periodically poled lithium niobate (PPLN) crystals doped with transition-metal ions by the Czochralski method, they accidentally found that samples doped with HfO_2_ show reasonable photorefractive resistance. Then the reduced photorefraction of hafnium-doped LiNbO_3_ (LN:Hf) were investigated [29,30]. It was found that the light-induced birefringence changes of LiNbO_3_ crystal doped with 4 mol% of HfO_2_ were comparable to that of 6 mol% MgO-doped crystals. This phenomenon indicates that Hafnium is another new damage-resistant element. Such a decrease of photorefraction is correlated to a corresponding increase in both the photoconductivity and the dark conductivity of the LN:Hf crystals. But it is strange that the dark conductivity of the 1 mol% HfO_2_ doped PPLN is larger than its photoconductivity. The distribution coefficient of Hf for 4 mol% HfO_2_-doped LiNbO_3_ is 0.93, which is nearer to one than that of Mg (1.2), so high quality LN:Hf crystal may be easier to grow than the usually used LiNbO_3_ crystal doped with 5 mol% MgO [31]. Later, more experiments of birefringence, second-harmonic phase-matching conditions and nonlinear coefficient were performed for a set of LN:Hf crystals as functions of dopant concentration. The data highlight that the doping threshold of Hf in LiNbO_3_ should be among 2.0 and 2.5 mol% in the melt [32,33]. The basic properties of LN:Hf have been investigated gradually, such as the Raman scattering spectroscopy, proving that Hf ions go into the Li-sites in the low doping concentration range and occupy both Li-sites and Nb-sites in high concentration [34]. Up to 11 mol% HfO_2_ doped LiNbO_3_ crystals were grown, but the good crystalline quality with a structural disorder increased linearly with increasing Hf content [35], and the electro-optic and dielectric properties were only slightly affected by the introduction of Hf ions [36,37].

**Figure 1:**
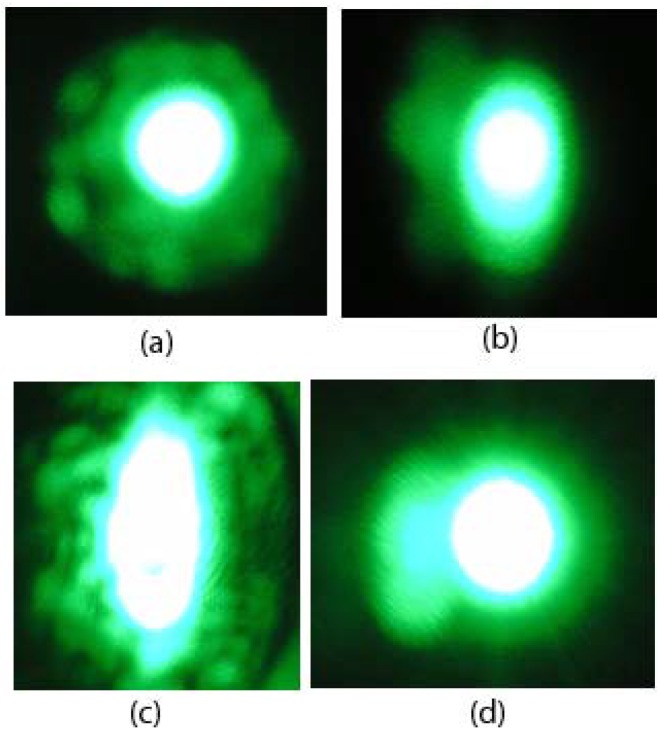
Distortion of Transmitted Argon Laser Beam Spots after 5 min of Irradiation. (**a**,**b**,**c**) LN:Zr1.7; (**d**) LN:Zr2. The light intensities are (**a**) 1.3 × 10^3^ W/cm^2^; (**b**) 1.3 × 10^4^ W/cm^2^; (**c**,**d**) 2.0 × 10^7^ W/cm^2^ [38].

In 2007, Kong *et al.* reported the outstanding optical damage resistance of zirconium doped LiNbO_3_ (LN:Zr) [38]. As shown in Figure 1, 2.0 mol% ZrO_2_-doped LiNbO_3_ can withstand a light intensity of 514.5 nm laser as high as 2.0 × 10^7^ W/cm^2^, which is more than 40 times larger than that of 6.5 mol% MgO-doped LiNbO_3_. Moreover, the doping threshold of Zr is near 2.0 mol%, where the distribution coefficient is about 0.97. In the same year, Sun *et al*. also reported that the saturated value of the birefringence change of 6 mol% ZrO_2_ doped LiNbO_3_ crystal is seven times smaller than that of congruent pure LiNbO_3_, but they found that the threshold concentration of the Zr^4+^ ions in LiNbO_3_ is about 6 mol% in the melt from the results of UV-Visible and infrared absorption spectra of LN:Zr crystals [39]. Argiolas *et al.* noticed the contradictory data, prepared a series of LN:Zr crystals with various Zr content, studied them using high-resolution x-ray diffraction and birefringence measurements as a function of the dopant content in the melt and concluded that the measured threshold value is very close to 2.0 mol% [40]. And, NSLN:Zr crystals can withstand a light intensity of 80 GW/cm^2^ at 532 nm pulse laser (the pulse duration 10 ns with a repetition rate of 1 Hz), which is close to that of KDP [41]. The overall investigation clearly indicates that LN:Zr could represent the optimal choice for the realization of room-temperature devices, such as Electro-optical modulators and all-optical wavelength converters [42].

In 2010 Wang *et al.* [43] reported that tin doped LiNbO_3_ (LN:Sn) crystals have a similar optical damage resistance as LN:Mg crystals, but the doping threshold of SnO_2_ is only 2.5 mol% and the distribution coefficient is close to one. As compared with other tetravalent ions doped LN, LN:Sn has a similar optical damage resistance as LN:Hf but a lower one than LN:Zr, Sn has a similar distribution coefficient as Zr and is closer to 1.0 than Hf with the doping concentration just near the threshold and dropping faster than Zr and Hf when it is above doping threshold. These experimental results suggest that Sn^4+^ is a new tetravalent ion that greatly increases the optical damage resistance of LN. 

The optical damage resistance of LN:Hf, LN:Zr and LN:Sn is collected in Table 1. For comparison, LN:Mg is also listed. We can see that LN:Zr has a much higher optical damage resistance as compared with other doped crystals. It is known that LN:Zr crystal has a higher photoconductivity, but further interpretation is still not clear.

**Table 1:** Optical Damage Resistance of LN:Mg, LN:Hf, LN:Zr and LN:Sn. The data, except for the doping threshold, come from 6.5 mol% MgO, 4.0 mol% HfO_2_, 2.0 mol% ZrO_2_ and 2.5 mol% SnO_2_ doped congruent LiNbO_3_ crystals, respectively.

Crystals Properties	LN:Mg	LN:Hf	LN:Zr	LN:Sn
Optical damage resistance (W/cm^2^, 514.5 nm)	5.0 × 10^5^	5.0 × 10^5^	>2.0 × 10^7^	4.8 × 10^5^
Saturation refractive index change (514.5 nm)	7.8 × 10^−6^	8.4 × 10^−6^	7.1 × 10^−7^	7.6 × 10^−6^
Doping threshold (mol% in melt)	4.6	2.0~2.5	2.0	2.5
Distribution coefficient	1.2	0.93	0.97	0.98
Optical damage resistance (W/cm^2^, 351 nm)	–	1.8 × 10^4^	1.6 × 10^5^	–
References	[19,31]	[31,33,56]	[38,55]	[43]

## 3. The Ultraviolet Photorefraction of Tetravalent Ions Doped LiNbO_3_

The ultraviolet (UV) photorefraction of nominally pure congruent LiNbO_3_ (CLN) was reported in 1992 [44]. Later, Laeri *et al.* made a review on UV photorefraction in various ferroelectrics in 1995 [45]. They reported that the photorefraction of pure LiNbO_3_, because of the diffusion mechanism, is enhanced in UV as compared to that in visible and that the dominant charge carriers in UV are holes. In 2000 Xu *et al.* [46] reported the strong UV photorefraction in highly Mg-doped LiNbO_3_ crystals with high two-wave mixing gain, fast response and low noise. They demonstrated experimentally that so-called damage-resistant dopants, such as Mg, are damage resistant only in the visible spectrum and that they will enhance photorefraction in the UV spectrum. In 2004, Qiao *et al.* [47] reported that the UV photorefraction of LiNbO_3_ doped with Zn and In was enhanced significantly as compared to that of the nominally pure LiNbO_3_ (PLN). They also stated that the resistance against photorefraction in highly Zn and In doped LiNbO_3_ is correct only in the visible light range. In highly Zn or In doped LiNbO_3_ crystals, diffusion dominates over photovoltaic effect, and electrons are determined to be the dominant charge carriers in UV photorefraction. This is inconsistent with what was reported by Jungen and Laeri [44,45]. The CTVE (charge transfer vibronic excitons) model, proposed by Vikhnin *et al*. [48], was utilized to interpret the enhancement of highly Zn and In doped LiNbO_3_, but the dominate charge carriers were not discussed. 

In fact, congruent LiNbO_3_ is far from the stoichiometric composition. There is plenty of intrinsic defects. The deficiency of Li_2_O induces Li-site vacancies (V_Li_^−^) and charge compensated anti-site Nb^5+^ (Nb_Li_^5+^) ions [49]. Part of the Nb_Li_^5+^ ions form small polarons (Nb_Li_^4+^) by trapping electrons, and some Nb_Li_^5+^ ions with neighboring normal-site Nb ions (Nb_Nb_^5+^) form bipolarons (Nb_Li_^4+^Nb_Nb_^4+^) by trapping a pair of electrons with opposite spins [50]. Bipolarons and small polarons can act as photorefractive centers and transform into each other by illumination with suitable light or with heat treatment [51,52]. And, in the visible region, the photorefractive centers of PLN were proven to be bipolarons and small polarons [53]. Hesselink *et al.* have utilized these bipolarons and small polarons to achieve nonvolatile holographic storage in PLN [54]. In UV photorefraction, we can consider that when LiNbO_3_ is illuminated by high energy UV light, electrons are excited from deep centers, e.g., Li vacancy or O^−^, to the conduction band, and holes are easily created because of the narrow gap between these deep centers and the valence band. Because there are plenty of Nb_Li_^5+^ ions in a CLN crystal, the electrons in the conduction band are easily captured by Nb_Li_^5+^ ions to form small polarons, and then bipolarons. However, the holes have no such affect, so in this situation, they may become the dominate charge carriers. For highly doped LiNbO_3_ crystals, when the doping concentration exceeds the threshold value, there is no Nb_Li_^5+^ ion, that is, no intrinsic defect, to capture the excited electrons by UV light, and then electrons become the dominate charge carriers because of their higher mobility than holes. Of course, further investigation is greatly required to fully understand this phenomenon.

**Figure 2:**
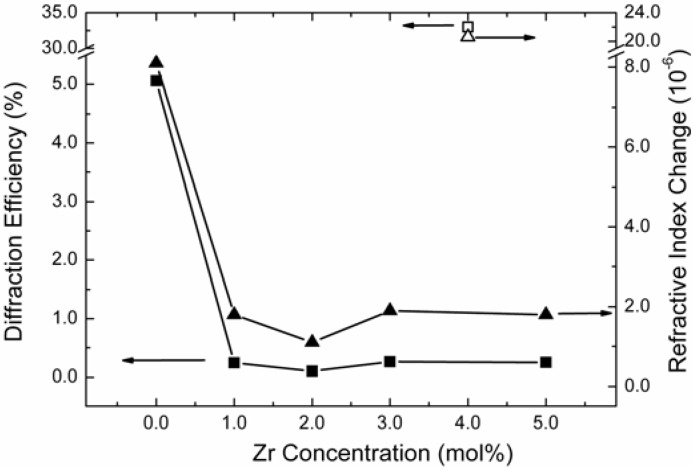
The Dependence of UV Photorefractive Diffraction Efficiency and Saturated Refractive Index Change of LN:Zr on the Doping Concentration of Zr. For comparison, the open symbols show the data for LN:Mg5. The lines are the guides for the eyes [55].

In 2010 Liu *et al.* [55] reported that LN:Zr crystals have high resistance against photorefraction in the UV region, as well as in the visible range, and can withstand a light intensity of above 10^5^ W/cm^2^ at 351 nm. The dependence of saturated diffraction efficiency on Zr concentration in LiNbO_3_ is shown in Figure 2. The saturated diffraction efficiency is apparently reduced when adding Zr into LiNbO_3_. The saturated refractive index change of LN:Zr2 is only 1.1×10^−6^, which is about eight times smaller than that of PLN, and only one twentieth of that of LN:Mg5. This experimental result indicates that LN:Zr is an excellent candidate for optical damage resistance from the UV to the visible spectrum. Furthermore, it was observed that the UV photorefractive gratings can be completely erased by 532 nm laser. This is quite different from the observation in the visible region, where reading with light of longer wavelength does not erase the photorefractive grating recorded by a shorter one because its photonic energy isn’t high enough to excite electrons from deep energy levels [13]. Therefore, they considered that this erasing process may have a relationship with another kind of charge carrier: holes; that is, 532 nm cannot excite electrons to the conduction band but can excite holes to the valence band. It is this process that erases the grating. It was thought that the UV photorefraction of highly doped LiNbO_3_ has a direct relationship with the dopants occupying Nb-sites as the doping concentration exceeds threshold value. The different performance of LN:Zr compared with LN:Mg and CLN was proposed. Since O^2−^ ions near Zr^4+^ and Mg^2+^ have different energy levels as the doping ions occupying Nb-sites, the energy level of O^2−^−Mg_Nb_^3−^ is shallower and closer to the photonic energy of 351 nm light than that of O^2−^–Zr_Nb_^−^. Therefore, 351 nm laser is more effective with LN:Mg but not with LN:Zr. 

**Figure 3:**
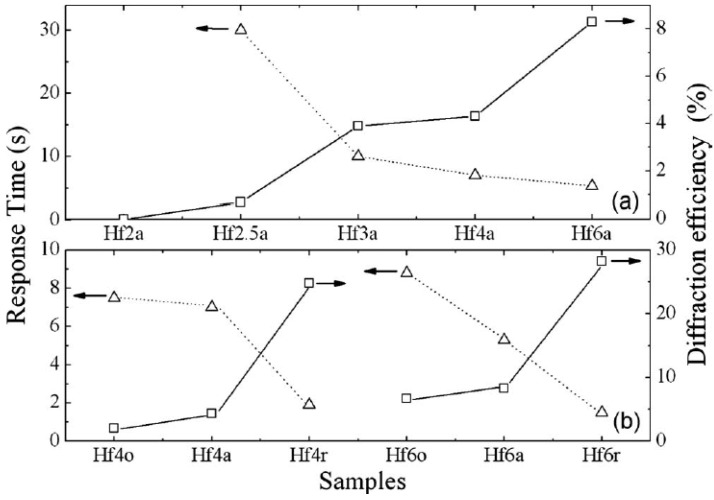
The Ultraviolet Photorefraction of LN:Hf Crystals at 351 nm. The samples are named after their Hf concentration in mol% and their as-grown (a), oxidized (o), or reduced (r) state [56].

However, the UV photorefractivity at 351 nm of LN:Hf is different from that of LN:Zr [56]. Figure 3a clearly demonstrates that Hf doping can improve the UV photorefractivity of LiNbO_3_, *i.e.*, higher diffraction efficiency and response speed can be obtained by Hf doping. Moreover, it is shown in Figure 3b that reduction can also induce a large enhancement of the UV photorefractivity. The energy transfer direction of two-wave coupling was proven to point to the -*c* end, and the reduction was found to enhance the UV photorefractivity. These results reveal the dominance of electrons in the UV photorefraction. Moreover, it was found that the grating recorded in highly Hf-doped LiNbO_3_ is nearly stable in the dark, but sensitive to red light. As shown in Figure 2 and Figure 3a, the photorefractive performance of LN:Hf and LN:Zr is different; LN:Hf has a higher saturated diffraction efficiency. If the datum of CLN in Figure 2 were drawn in Figure 3(a), we could see that when the doping concentration of Hf increases, the saturated diffraction efficiency will drop to the minimum at LN:Hf2a, then increase gradually. This phenomenon is just like the situation of LN:Zr, where the diffractive efficiency increases very slowly. This result indicates that the diffractive efficiency decreases fast when the doping concentration of tetravalent ions is below its threshold, reaches the minimum at the doping threshold, and then increases with increasing doping concentration. The different saturated efficiency between LN:Hf and LN:Zr may be due to the different energy of O^2−^–Hf_Nb_^−^ and O^2−^–Zr_Nb_^−^. 

The photorefractivity at 325 nm of LN:Hf and Hf:Sn were also investigated [57,58]. It was found that the UV photorefraction of LN:Hf was enhanced significantly as compared to that of PLN. This result clearly indicated that the property of resistance against photorefraction in highly Hf doped LiNbO_3_ was true only in the visible and near-infrared wavelength range. LN:Hf exhibited excellent photorefractive characteristics at a UV wavelength of 325 nm, even better than those at 351 nm. The diffraction efficiency, the holographic recording sensitivity and response rate, and the two-wave coupling gain coefficient are greatly enhanced when the Sn-doping concentration reaches 2.0 mol% or more. Unlike LN:Hf in which the UV gratings can be erased easily by a red beam, the UV gratings in LN:Sn can withstand long-term red beam illumination. For LN:Hf and LN:Sn crystals, diffusion dominates over photovoltaic effect, and electrons are the dominant charge carriers in UV photorefraction at 325 nm. Now, further investigation is greatly needed to clarify the photorefractive characteristics of LiNbO_3_ near the absorption edge.

## 4. The Photorefraction of Tetravalent Ions Co-Doped LiNbO_3_

In 2006 Li *et al.* [59] reported the photorefractive properties of LiNbO_3_ co-doped with HfO_2_ and Fe_2_O_3_ (LN:Hf,Fe). Then the defect structure, spectroscopy and optical properties were investigated [60,61,62]. Dissimilar to Mg^2+^ (or Zn^2+^, In^3+^) and Fe co-doped LiNbO_3_ crystals [63,64], Fe ions still remain at Li sites in LiNbO_3_:Hf,Fe crystals when the HfO_2_-doping concentration goes above its threshold value. As a result, the response rate and sensitivity are greatly improved. Meanwhile, the saturation diffraction efficiency remains at a high value. Therefore, hafnium ion was considered as an efficient ion to improve the photorefractive properties of LN:Fe crystal. The saturation diffraction efficiency could reach 80.2% in the LN:Hf,Fe crystal grown from the melt with the [Li]/[Nb] ratio of 1.20 [65]. And the gain coefficient of LN:Hf,Fe increases dramatically with temperatures up to 110 °C [66]. The similar phenomenon was also found for Hf and Ce co-doped LiNbO_3_ (LN:Hf,Ce). It was reported that the photorefractive properties of LN:Ce crystals were improved by HfO_2_ co-doping, and oxidation/reduction treatment had a great effect on the photorefractive properties [67].

Later, Kong *et al.* [68] reported the photorefractive characteristics of LiNbO_3_ crystals co-doped with ZrO_2_ and Fe_2_O_3_ (LN:Zr,Fe). Compared with LN:Hf,Fe, although Zr^4+^ and Hf^4+^ are all tetravalent ions, the concentration of Fe^2+^ ions in as-grown LN:Zr,Fe is much higher than that in LN:Hf,Fe. As a result, the photorefractive response speed of these as-grown LN:Zr,Fe crystals is only 2 s and the sensitivity is larger than 12 cm/J, while the saturation diffraction efficiency still remains at a high level. Therefore, zirconium ions are a preferable choice to improve the photorefractive properties of LN:Fe crystal. It was found that as the [Li]/[Nb] ratio increases, the distribution coefficients of Fe and Zr ions decrease, the absorption edge shifts to a shorter wavelength and the threshold concentration of ZrO_2_ decreases [69]. Fan *et al.* reported that NSLN:Zr,Fe crystals have significantly enhanced photorefractive response speed, recording sensitivity and two-wave coupling gain coefficient as compared with CLN:Zr,Fe, while the high saturation diffraction efficiency is still maintained [70]. But Luo *et al.* reported that the photorefractive properties of LN:Zr,Fe decrease with increasing [Li]/[Nb] ratios [71]. Further investigation is needed to clarify the function of [Li]/[Nb] ratio on the photorefractive characteristics of LN:Zr,Fe crystals.

**Figure 4:**
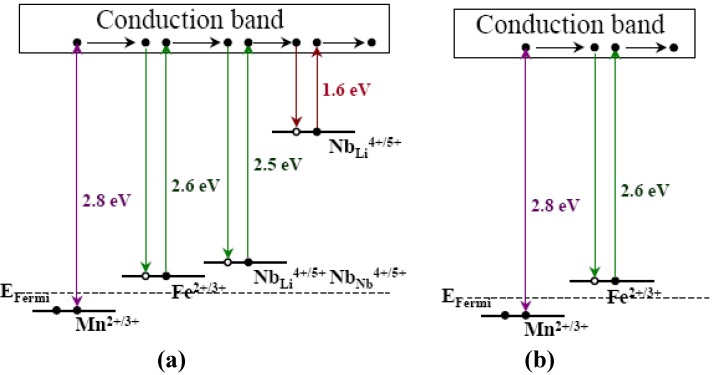
Energy level diagram of dopants and intrinsic defects in the forbidden gap of LiNbO_3_. (**a**) Normal congruent LiNbO_3_, doubly doped with iron and manganese, where the energy levels of small polaron (Nb_Li_^4+^) and bipolaron (Nb_Li_^4+^Nb_Nb_^4+^) also exist; (**b**) Ideal doubly doped LiNbO_3_ [72].

Then, Kong *et al.* [72] designed Zr, Fe, and Mn triply doped LiNbO_3_ (LN:Zr,Fe,Mn) crystals. They analyzed the energy level diagram of LN:Fe,Mn reported by Buse *et al.* [13] and considered that in commercial CLN codoped with Fe and Mn, the energy levels of Fe^2+/3+^, Mn^2+/3+^, Nb_Li_^4+^Nb_Nb_^4+^ and Nb_Li_^4+^ should at least be included, as shown in Figure 4a. The intrinsic photorefractive centers slow down the electron mobility for non-volatile storage appreciably. Therefore, in order to increase the sensitivity of LN:Fe,Mn crystal, intrinsic defects should be eliminated such that a real two energy level system is obtained as shown in Figure 4b. The results of LN:Hf,Fe and LN:Zr,Fe have shown that when tetravalent ions are co-doped into LN:Fe, they only push Nb_Li_^5+^ ions to Nb-sites and do not affect the site occupation of Fe^2+/3+^ ions, thus resulting in a significant shortening of the response time [59,68]. As Mn^2+/3+^ ions have the same valence as Fe^2+/3+^ ions, all lower than +4, it is reasonable to assume that tetravalent ions do not affect the site occupation of Mn^2+/3+^ ions. The experimental results proved that the response time of LN:Zr,Fe,Mn for nonvolatile holographic storage became smaller than 1.0 s, and the measured sensitivity *S’* became as high as 1.31 cm/J [72]. LN:Zr,Fe,Mn crystal combines the advantages of high diffraction efficiency, long storage lifetime, fast response speed, strong resistance to light-induced scattering and non-volatility. Thus it seems to be an excellent recording medium for practical holographic storage devices. The spectroscopy, defect structure and optical characteristics of LN:Zr,Fe,Mn with different doping concentrations and [Li]/[Nb] ratios were investigated [73,74]. The high diffraction efficiency and significantly enhanced response speed and sensitivity were also reported at 488 nm laser [75]. The nonvolatile holographic storage of Hf, Fe and Mn triply doped LiNbO_3_ (LN:Hf,Fe,Mn) was investigated. The recording speed was faster with the increase of HfO_2_ doping concentration, and at the same time, little loss of nonvolatile diffraction efficiencies could be achieved [76].

**Figure 5:**
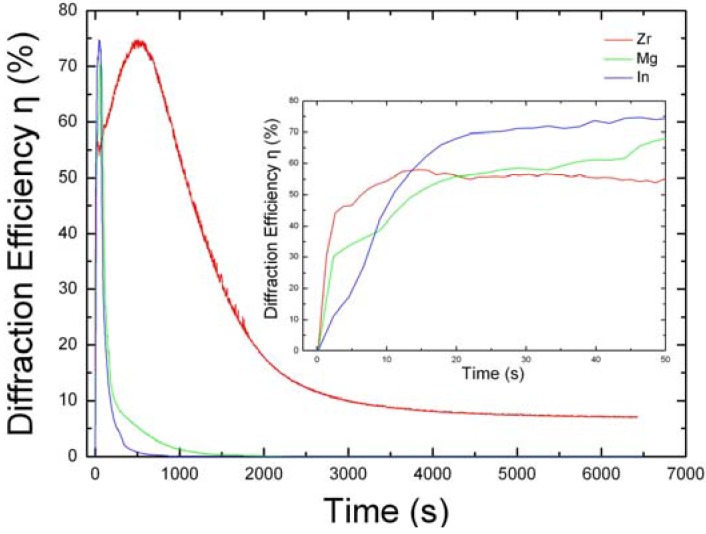
Holographic Recording and Fixing Characteristics of LN:Zr,Fe,Mn, LN:Mg,Fe,Mn and LN:In,Fe,Mn. Recording time is 50 s. Readout time is more than 100 min. The inset shows the recording process (0 s to 50 s).

It should be pointed out that the improvement of nonvolatile sensitivity in doped LiNbO_3_ is directly related to the valence of Zr and Hf if other optical damage resistant ions, such as Mg^2+^ and In^3+^, are codoped into LN:Fe,Mn. Because their valences are the same as Fe^2+/3+^ and Mn^2+/3+^, these codoping ions will affect the lattice occupation of Fe and Mn ions and let them lose their function as photorefractive centers. As shown in Figure 5, nonvolatile holographic storage cannot be accomplished. In fact, the no nonvolatile storage of LN:Mg,Fe,Mn had been found by Liu *et al.* when they investigated the nonvolatile holographic storage of four kinds of LiNbO_3_ crystals doped with Cu:Ce, Mn:Cu:Ce, Mn:Fe and Mn:Fe:Mg [77]. They found that on the condition of non-volatile holographic storage with high signal-to-noise ratio, the non-volatile diffraction efficiency of the oxidized LN:Cu,Ce crystal is the highest among all studied samples, and the recording sensitivity and the dynamic range of the oxidized LN:Mn,Fe crystal are the highest. Because the nonvolatile storage of LN:Mg,Fe,Mn was not accomplished, this phenomenon did not receive attention, and the micro-mechanism was not analyzed. 

Zr, Cu and Ce triply doped LiNbO_3_ (LN:Zr,Cu,Ce) crystals have also been designed and grown [78]. As compared with congruent LN:Cu,Ce crystal, the photorefractive sensitivity *S* of LN:Zr,Cu,Ce is exceptionally improved one order of magnitude [79], and the nonvolatile sensitivity *S’* is comparable to that of NSLN:Cu,Ce [17]. The light-induced scattering in LN:Zr,Cu,Ce is suppressed effectively, which is much lower than that in LN:Zr,Fe,Mn. Because triply doped LN:Zr,Cu,Ce has low light induced scattering and high photorefractive sensitivity, it is a suitable candidate for nonvolatile holographic storage. However, it should be pointed out that the photorefractive enhancement of Zr-codoping for LN:Cu,Ce is smaller than for LN:Fe,Mn. This might be related to the site occupation change of Ce ions when Zr ions were codoped into it. Because part of the Ce ions have the same tetravalence as Zr ions, when the concentration of Zr exceeds its doping threshold, a part of Ce^4+^ ions will be pushed to Nb sites from Li sites. These Ce^4+^ ions cannot trap electrons and lose their ability as photorefractive acceptors. As a result, the photorefraction caused by Ce^3+/4+^ centers is decreased and the nonvolatile diffraction efficiency is a little lower. The nonvolatile holographic storage properties of Hf, Cu and Ce triply doped LiNbO_3_ (LN:Hf,Cu,Ce) crystals were also studied [80]. The results indicated that the codoping with Hf eliminated undesirable intrinsic electron traps, which strongly enhanced the charge transition speed. As the doping concentration of Hf increased, the fixed diffraction efficiency decreased, but the holographic response time and photorefractive sensitivity improved.

Another tetravalent ion Ru-doped LiNbO_3_ (LN:Ru) was noted for its nonvolatile recording properties [81]. In spite of the single doping, a nonvolatile hologram can be recorded at a wavelength of 633 nm when a blue light is simultaneously incident on the crystal. The diffraction efficiency of the nonvolatile hologram is about 1% at the extraordinary polarization, but this crystal shows only a small loss of the hologram strength at the beginning of the readout process. An absorption spectra examination of LN:Ru showed that there were two absorption peaks around 370 and 530 nm [82]. The absorption coefficients should increase and the absorption edges shift toward longer wavelength as the Ru concentration increases. The OH^−^ absorption spectra confirm that Ru ions may have substituted for both Li vacancies and antisite Nb ions, as the Ru doping concentration in the present case is well below the threshold limit. The dark decay time constant of the LN:Ru decreased as the temperature increased, from which it can be deduced that in lithium niobate, the energy level is shallower at the Ru center than that at the Fe center [83]. 

One way to improve the recording properties of LN:Ru is codoping Fe ions together with Ru ions (LN:Ru,Fe) [84]. With this approach, the sensitivity and the dynamic range were dramatically enhanced, such as a high sensitivity of 0.12 cm/J, and a large dynamic range of 0.53 were obtained in a 0.5 mm-thick crystal. To further improve the nonvolatile storage characteristics of LN:Ru,Fe crystals, Zr ions can be triply doped (LN:Zr,Ru,Fe) [85]. In oxidized LN:Zr,Ru,Fe crystal, a short response time of 9.1 s and a high recording sensitivity of 0.92 cm/J were demonstrated, together with a high fixed diffraction efficiency of 44.9% maintained. One problem of Ru doped LiNbO_3_ crystals in practical application is that the Ru concentration in the grown crystal gradually decreased along the pulling direction because the effective segregation coefficient of Ru in lithium niobate is greater than one, and also because RuO_2_ evaporated during the crystal growth period [82].

For a better overview, the photorefractive properties of LN:Hf,Fe, LN:Zr,Fe, LN:Zr,Fe,Mn, LN:Zr,Cu,Ce and LN:Zr,Ru,Fe are collected in Table 2. As a comparison, LN:Fe and LN:Mg,Fe are also listed. We only collected the typical data from the references, so different crystals may have different doping concentrations. 

**Table 2:** Photorefractive Properties of LN:Fe, LN:Mg,Fe, LN:Hf,Fe, LN:Zr, Fe, LN:Zr,Fe,Mn, LN:Zr,Cu,Ce and LN:Zr,Ru,Fe.

**Crystal**	**Doping concentration**					**Photorefractive properties**				**Reference**
	Fe_2_O_3_ (wt%)	MgO (mol%)		HfO_2_ (mol%)	ZrO_2_ (mol%)	*η_sat_* (%)	*τ_r_* (s)	*S* (cm/J)	*S’* (cm/J)	
LN:Fe	0.01					70	160			[23]
LN:Mg,Fe	0.01	6				15	15			[23]
LN:Hf,Fe	0.03			5		55.4	10.7	5.23		[59]
LN:Zr,Fe	0.03				2	32.0	1.8	12.87		[68]
LN:Zr,Fe,Mn	0.075	0.01 wt% MnO			2	56.4	0.95	3.47	1.31	[72]
LN:Zr,Cu,Ce	0.011 wt% CuO		0.085 wt% Ce_2_O_3_		2	62.4	120	0.312	0.099	[78]
LN:Zr,Ru,Fe	0.15 wt% Fe_2_O_3_		0.15 wt% RuO_2_		2	60.5	9.1		0.92	[85]

## 5. The Photorefraction of Pentavalent and Hexavalent Ions Doped LiNbO_3_

As pointed out in the Introduction, until now, iron was considered as the most effective dopant for the photorefraction of LiNbO_3_, though several problems, especially the low response speed, impede it from becoming a commercial recording medium. When analyzing the effects of currently used dopants, we noticed that their valences are all below 5+, the valence of Nb. These dopants, therefore, preferably occupy Li-sites. Then a marvelous thought arose: if LiNbO_3_ is doped with ions of valence 5+ or 6+, these ions may occupy Nb-sites, and thereby induce new effects.

**Figure 6:**
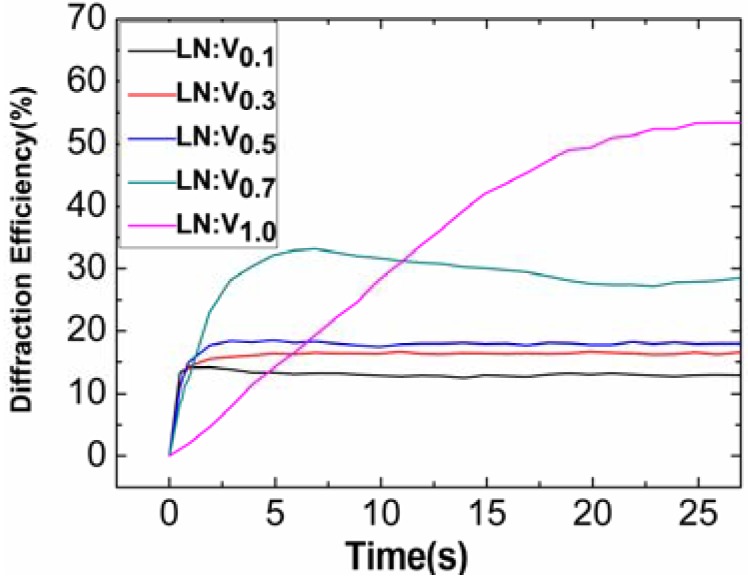
The time dependence of diffraction efficiency of LN:V crystals. The vanadium-doping concentrations are 0.1, 0.3, 0.5, 0.7 and 1.0 mol% for LN:V0.1, LN:V0.3, LN:V0.5, LN:V0.7 and LN:V1.0, respectively. The laser wavelength is 351 nm; the light intensities of the signal and reference beams are 283 mW/cm^2^ and 300 mW/cm^2^ [86].

Dong *et al.* [86] grew a series of vanadium-doped lithium niobate (LN:V) crystals and investigated their photorefractive properties. As shown in Figure 6, a fast photorefractive response of 0.16 s was obtained for 0.1 mol% V-doped LiNbO_3_ with a 351 nm laser and a total light intensity of 583 mW/cm^2^. The spectroscopic measurements show that V^3+^, V^4+^ and V^5+^ ions exist in these LN:V crystals. V^3+^ and V^4+^ ions correspond to the 420 nm and 475 nm absorption peaks respectively. The fast photorefractive response and high sensitivity indicate that LN:V is a competitive candidate for UV photorefraction. Even in the visible region, a short photorefractive response time of 0.57 s in LN:V0.1 at a total intensity of 471 mW/cm^2^ could be obtained [87]. The experimental results also show that electrons are the dominant charge carriers and that the diffusion field dominates over the photorefraction process. Because of its low doping concentration and absorption coefficient, the overall scenarios [86,87] indicate that LN:V may be a suitable material for holographic storage. From Figure 6, we can also see that the time dependence of 0.7 mol% and 1.0 mol% V-doped LiNbO_3_ is very different from the others; they have a slower response and a higher saturated diffraction efficiency. It was proposed that a doping threshold existed between 0.5 and 0.7 mol%, which may be caused by a site occupation change of V ions from Li-sites at lower concentrations to Nb-sites at higher concentrations, or by some complicated defect clusters forming at higher doping concentrations. Normally ions will lose their function as photorefractive centers when changing the site occupation from Li-site to Nb-site. However, related experiments are greatly needed to explore what happens when the doping concentration increases from 0.5 to 0.7 mol%.

**Figure 7:**
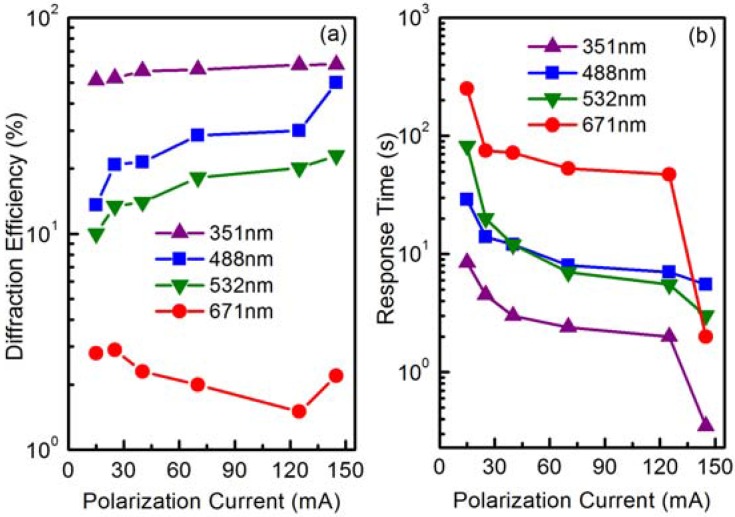
The UV-visible photorefractive characteristics of LN:Mo0.5 crystals polarized under various polarization currents for 15 min. The light intensity per beam is 320, 400, 400 and 3000 mW/cm^2^ for 351, 488, 532 and 671 nm lasers, respectively [88].

Tian *et al.* [88] grew molybdenum doped lithium niobate (LN:Mo) crystals under different polarization conditions and investigated their holographic properties. The XPS measurement identified the valence of the Mo ions in LiNbO_3_ as 4+, 5+ and 6+. X-ray single crystal diffraction analysis showed Mo^6+^ ions push regular Nb^5+^ to Li sites, forming new defects of Mo_Nb_^+^ and a larger amount of anti-site Nb^5+^. That is in contrast to current dopants; hexavalent molybdenum prefers niobium sites. As shown in Figure 7, holographic storage becomes possible from the ultraviolet to the visible spectrum with considerably lower response time. The response time of 0.5 mol% Mo doped LiNbO_3_ especially can be shortened to as small as 0.35 s with a still high saturation diffraction efficiency of about 60% at 351 nm. These features can be attributed to Mo^6+^ ions occupying regular Nb sites. The novel defect cannot be created by other dopants currently in use. LN:Mo is thus a promising candidate for all-color holographic storage applications.

## 6. Summary

The recent developments in the photorefraction of doped lithium niobate crystals are reviewed, especially the photorefractive performance of LN:Hf, LN:Zr, LN:Sn; LN:Hf,Fe, LN:Zr,Fe, LN:Zr,Fe,Mn, LN:Zr,Cu,Ce, LN:Ru, LN:V and LN:Mo. Among them, LN:Zr,Fe,Mn shows excellent nonvolatile holographic storage properties, and LN:V and LN:Mo have fast response speed and multi-wavelength storage characteristics. Has the research of dopants in LiNbO_3_ reached its plateau? Of course not. Just watch the bustling activities in the past several years. And, many aspects of these high valence ions doped LiNbO_3_ are still not clear. Dopants in LiNbO_3_ will continue to attract scientific and technical interest. The properties will have to be understood so that the dopants can either be eliminated or put to good use.
